# Efficacy and Outcome of Remdesivir and Tocilizumab Combination Against Dexamethasone for the Treatment of Severe COVID-19: A Randomized Controlled Trial

**DOI:** 10.3389/fphar.2022.690726

**Published:** 2022-04-05

**Authors:** Abu Taiub Mohammed Mohiuddin Chowdhury, Aktar Kamal, Kafil Uddin Abbas, Shubhashis Talukder, Md Rezaul Karim, Md. Ahsan Ali, Md. Nuruzzaman, Yarui Li, Shuixiang He

**Affiliations:** ^1^ Department of Gastroenterology, First Affiliated Hospital of Xi’an Jiaotong University, Xi’an, China; ^2^ Ministry of Health and Family Welfare (OSD-DGHS), Dhaka, Bangladesh; ^3^ Department of Critical Care, M Abdur Rahim Medical College Hospital, Dinajpur, Bangladesh; ^4^ Department of Critical Care, Cox’s Bazar 250 Bed District Sadar Hospital, Cox’s Bazar, Bangladesh; ^5^ Department of Critical Care, 250 Bed Chattogram General Hospital, Chattogram, Bangladesh; ^6^ Hubei Key Laboratory of Embryonic Stem Cell Research, Institute of Neuroscience, Hubei University of Medicine, Shiyan, China; ^7^ Acute Medical Unit, University Hospital Limerick, Limerick, Ireland; ^8^ Department of Histology, Xi’an Jiaotong University, Xi’an, China; ^9^ Department of Internal Medicine, M Abdur Rahim Medical College Hospital, Dinajpur, Bangladesh

**Keywords:** COVID-19, SARS-Cov-2, remdecivir, tocilizumab, dexamethasone, COVID-19 ARDS, Bangladesh

## Abstract

**Objective:** In this study, we investigated the efficacy and safety of remdesivir and tocilizumab combination therapy against dexamethasone for the management of severe COVID-19 patients.

**Methods:** This was a multicenter study. Cases were randomly chosen and divided into two groups using an odd–even ratio of 1:1 applied to the hospital registration number. Group A received remdesivir [5 mg/kg (<40 kg) or 200 mg (>40 kg) on day 1 and then 2.5 mg/kg (<40 kg) or 100 mg (>40 kg) daily] + tocilizumab [8 mg/kg up to 800 mg highest 12 h apart], and group B was the control and received dexamethasone 6 mg/day. In addition, a broad-spectrum antibiotic and other essential treatments were received by all patients. To evaluate the mortality risk, the sequential organ failure assessment (SOFA) score was calculated on day-1. Treatment outcomes were measured as time to clinical improvement; mortality rate; duration of ICU stay; total period of hospitalization; the rate of (Supplementary Material) oxygen use; time to clinical failure; National Early Warning Score-2 (NEWS), and the percentage of lung recovery on CT of chest on discharge. Clinical trial registration ID: NCT04678739.

**Results:** Remdesivir-Tocilizumab group had a lower mortality rate (25.49%) than the control (30.77%). The time to clinical improvement (Group A-9.41; B-14.21 days), NEWS-2 on discharge (Group A-0.89; B-1.2), duration of ICU stay (Group A-7.68; B-10.58), and duration of hospitalization (Group A-9.91; B-14.68) were less in the treatment group. Group A had a better percentage of lung recovery on chest CT than the control (Group A-22.13; B-11.74). All these differences were statistically significant (*p*= <0.05) in a *t*-test. However, no significant survival benefit was found among the study groups in Kaplan–Meier survival analysis, *p* = 0.739.

**Conclusion:** The remdesivir–tocilizumab combination had preferable outcomes compared to the dexamethasone therapy for the treatment of severe COVID-19 concerning mortality rate and clinical and pulmonary improvement, although it did not demonstrate a significant survival benefit.

**Clinical Trial Registration:** https://clinicaltrials.gov, NCT04678739.

## Introduction

The coronavirus disease 2019 (COVID-19) was first reported in China and then quickly spread all over the world. (Velavan and Meyer, 2020) This was formerly recognized as the 2019‐nCoV. Tyrell and Bynoe were the first to isolate and describe coronaviruses in 1966. Coronavirus is an RNA virus and possesses the ability to infect humans and animals. (Tyrrell and Bynoe, 1966) SARS‐CoV‐2 is a member of the beta-corona virus family and contains a similar genome to a bat coronavirus. (Zhouyxwxea et al., 2020) COVID-19 has posed frequent challenges during its course, ranging from virus isolation to detection and prevention. It causes many human respiratory tract infections (RITs), varying from a mild cold to severe respiratory distress syndrome. (Heymann and Shindo, 2020) The COVID-19 treatment mostly depends on the patient’s clinical features and severity of the disease. This might include antivirals, corticosteroids, low molecular weight heparins (LMWHs), convalescence plasma, and immunoglobulins, though until now, no treatment can act specifically against COVID-19. (Stasi et al., 2020) SARS-CoV-2 is a highly infectious virus that causes injury to the pulmonary alveolar epithelium and results in acute respiratory distress syndrome or ARDS. Therefore, for the early identification and proper management, it is crucial to understand the features of COVID-19–induced ARDS. (Gibson et al., 2020) (Li and Ma, 2020) COVID-19 is a disease characterized by pneumonia of viral origin, a hyperimmune and hypercoagulation state, and till now there is no definitive therapy against this condition. All the international medical societies suggest supportive care and therapy, especially discouraging the use of corticosteroids and antibiotics for the management of COVID-19. All the initial studies of COVID-19 reported supportive care management and showed a high death rate with a long ventilator support duration (Kory et al., 2021). Though, mostly the antiviral agents remdesivir and favipiravir and immunomodulatory agents are in use, other drugs such as HCQ (hydroxychloroquine), chloroquine, and recently ivermectin have been prescribed for the mild-to-moderate type of COVID-19 infection. (Chowdhury, 2021) Though, the NIH (National Institute of Health) has discouraged the random use of antiviral drugs, COVID-19 management includes patient isolation, universal supportive care, respiratory, symptomatic, nutritional, and psychosomatic management (Liu and Liu, 2020a) (Panghzsea et al., 2020). According to the current state of knowledge and based on the literature reports, exploring possible antivirals and associated drugs that act against inflammatory conditions that can help fight against COVID-19–ARDS in severe COVID-19 patients is very important. But, till now, though outcomes had been reported separately regarding an antiviral such as remdesivir and an anti–interleukin-6 receptor monoclonal antibody, such as tocilizumab, no study has been performed regarding the combined efficacy of remdesivir and tocilizumab on severe COVID-19 patients. In this randomized controlled trial (RCT), we investigated the efficacy and outcome of the remdesivir and tocilizumab combination, in comparison to dexamethasone, for the treatment of severe COVID-19–ARDS. Our objective was to find and establish a better treatment option for severe COVID-19.

## Methodology

This was a multicenter study carried out in the COVID-19 Special Care and dependency unit of the M. Abdur Rahim Medical College Hospital, Cox’s Bazar 250-bed Sadar Hospital, and Chattogram General Hospital, Bangladesh. The estimated sample size was 384. This was calculated by using the following formula: n = z^2^pq/d^2^, where z = at 95% confidence limit the value of z is 1.96; n = required sample size, *p* = estimated prevalence = 0.5, q = 1-p, and d = margin of error at 5% (standard value of 0.05).

## Inclusion and Exclusion Criteria

COVID-19 cases with positive RT-PCR for SARS-CoV-2 infection who required admission in the ICU with a National Early Warning Score-2 (NEWS-2) ≥5 were randomly chosen and included in the study. Then, they were divided into two study groups (A and B) by using an odd–even ratio of 1:1 applied to the hospital registration number. After enrollment, every case was further accessed and confirmed by the investigators or attending physician. Acute respiratory distress syndrome (ARDS) was identified according to the Berlin definition.

Patients who had a pre-existing uncontrolled chronic condition with major compromised organ function before COVID-19 (severe ischemic heart disease, epilepsy, advanced pulmonary/renal/hepatic disease, corpulmonale, long-standing severe uncontrolled DM, etc.), those who were immunocompromised, those with an advanced stage of carcinoma, in chemo or adjuvant therapy, pregnant patients, those with AIDS, pulmonary tuberculosis, those with contraindications/possible drug interactions to remdisivir/tocilizumab/dexamethasone, and those who were already hospitalized at the time of enrollment due to other causes were excluded from the study.

**FIGURE 1:**
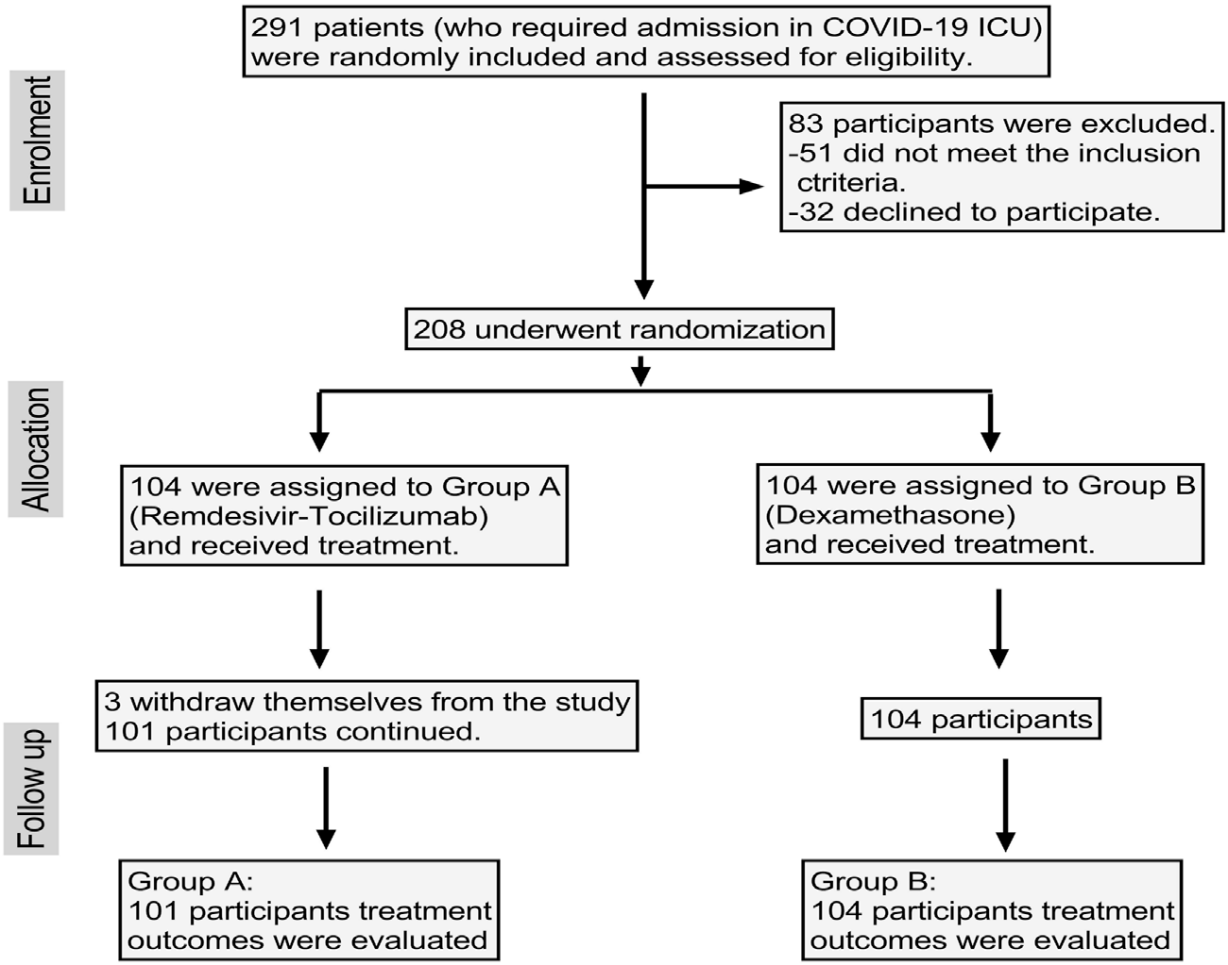
Flow diagram.

Initially, 291 patients were enrolled, among which 32 were unwilling to participate and 51 patients had pre-existing comorbid conditions or were hospitalized at that time due to other reasons and so were excluded. That left 208 patients enrolled, though three patients in group A withdrew themselves from the study. Finally, data of 205 patients were evaluated (Figure 1).

## Study Groups and Treatment Outcome

Group A (n = 101) received remdesivir [5 mg/kg (<40 kg) or 200 mg (>40 kg) on day 1 and then 2.5 mg/kg (<40 kg) or 100 mg (>40 kg) daily]+ tocilizumab [8 mg/kg up to 800 mg highest 12 h apart]; and Group B (n = 104), the control group, received dexamethasone (6 mg/day in divided dose).

In addition, a broad-spectrum antibiotic (meropenem), PPI, ascorbic acid, cholecalciferol, zinc, and symptomatic treatments were received by all patients.

Treatment outcomes were measured as follows: time to clinical improvement, defined as the time from randomization to NEWS-2 score (National Early Warning Score 2) (Morgan, 1997) of ≤2 as maintained for 24 h; mortality rate; duration of ICU stay; total period of hospitalization; the rate of (Supplementary Material) oxygen use; time to clinical failure; NEWS-2 score on discharge; and the percentage of lung recovery on CT of the chest (the difference value of the CT involvement of lung pathology during admission and at the time of discharge). Sequential organ failure assessment (SOFA) scores were calculated to evaluate the mortality risk on the admission day. Routine follow-ups were obtained at an interval of every 24 h.

## Ethics and Consent

The purpose of the study was properly explained to the patients, and written informed consent was obtained in every case. This study was approved by the ethical approval committee of M. Abdur Rahim Medical College, Approval No: M.A.R.M.C.D/2020/1985. Clinical trial registration: NCT04678739.

## Statistical Analysis

Statistical analysis was carried out by SPSS version 27 and GraphPad prism 7. Each group was analyzed to calculate the mean ± SD and mean ± SEM. Frequency was calculated. T-tests, chi-square tests, and Kaplan–Meier survival analysis were performed among the groups to evaluate the statistical significance. Required groups were calculated in percentage and compared.

## Results

### General Demographics and the Treatment Outcome in the Study Groups

The total number of patients was 205 (M. Abdur Rahim Medical College Hospital- 95, Cox’s Bazar 250-bed Sadar Hospital- 63, and Chattogram General Hospital- 47); men 156 (76.09%) and women 49 (23.90%); group A 101 (25women, 76men, and mean age 56.64 years) and group B 104 subjects (24 women, 80men, and mean age 57.04 years) (Table 1). The highest affected group in our study was the 61–70 years age group, 23.41% (48), and the lowest was the 10–20 years group, 2.4% (Stasi et al., 2020). In the case of group A, this trend was 51–60 and 61–70 years; in group B, the age groups were 41–50 years and 61–70 years (Table 4).

**TABLE 1:** Patient demographics, characteristics, and treatment outcomes among the study groups. Data presented as mean ± SD.

	Variables	Group A	Group B (Control)	*T*-test (95% CI)
	Gender	*n* = 101; Male-76 (73.1%); Female-25 (24%)	*n* = 104; Male-80 (76.93%); Female-24 (23.07%)	
Patient demographics and characteristics of study group patients during hospitalization.	Age (In years) 56.64 ± 15.05; 18–85 years	56.24 ± 15; 18–85 years	57.04 ± 15.15; 23–83 years	*p* = 0.259
	Body weight (Kg)	65.4 ± 9.0 (51–94)	66.5 ± 7.7 (54–91)	*p* = 0.61
	BMI	23.6± 5.6 (19.8–36.4)	22.9 ± 5.8 (21.4–35.7)	*p* = 0.58
	Comorbidity	61 (58.7%)	46 (44.2%)	
	NEWS-2 Score (On admission)	8.267 ± 1.918; 5–12	8.788 ± 2.037; 5–13	*p* = 0.060
	SOFA (Sequential organ failure assessment) day-1 score	6.06 ± 1.67; 3–11	5.35 ± 1.58; 3–11	*p* = 0.021**
	Oxygen saturation (%)	85.77 ± 8.971; 34–99%	85.23 ± 6.961; 73–98%	*p* = 0.0243*
	PaO2 (mm of Hg)	46.09 ± 9.8; 23–83	47.91 ± 6.8; 38–91	*p* = 0.005**
	P:F ratio (On admission)	90.48 ± 39.6; 23–199	75.63 ± 41.79; 38–220	*p* = 0.0093**
	Oxygen requirement	19.44 ± 16.56; 4–60 L/min	18.38±10.51; 2–40 L/min	*p* = 0.643
	CT chest %	47.33 ± 19.4; 15–95%	32.15 ± 17.51; 0–65%	*p* = <0.0001****
	Respiratory rate	29.07 ± 10; 12–55/min	30.37 ± 6.865; 18–45/min	*p* = 0.299
	Temperature	100.7 ± 1.865; 98–104°F	101.4 ± 1.453; 98–104°F	*p* =0.398
	Serum Creatinine	1.14 ± 0.8; 0.8–3	0.98 ± 0.7; 0.6–3	*p* = 0.118
	Serum Bilirubin	1.62 ± 1.07; 0.4–5.3	1.52 ± 0.9; 0.4–5	*p* = 0.19
	Platelet count (10^3^ /ml)	207.5 ± 92.8; 75–400	224.4 ± 91.2; 87–400	*p* = 0.19
	MAP(Mean arterial pressure in mm of Hg)	86.16 ± 13.55; 54–120	86.26±12.19; 54-116	*p* = 0.95
	GCS Score	12.19 ± 1.79; 7–15	12.67 ± 1.93; 7–15	*p* = 0.064
Characteristics of study group patients during discharge.	NEWS-2 Score (On discharge; Recovered cases)	0.89 ± 0.84; 0–2	1.22 ± 0.87; 0–2	*p* = 0.0221*
	Oxygen saturation (On discharge; Recovered cases)	95.91 ± 2.07; 90–100%	96.35 ± 1.558; 93–99%	*p* = 0.080
(Recovered cases)	Oxygen requirement (On discharge; Recovered cases)	1.36 ± 2.288; 0–10 L/min	1.569 ± 2.42; 0–12 L/min	*p* = 0.279
	CT chest % (On discharge; Recovered cases)	17.67 ± 8.193; 0–35%	15.35 ± 10.07; 0–38%	*p* = 0.127
	Respiratory rate (On discharge; recovered cases)	19.4 ± 4.74; 12–34/min	19.11 ± 2.17; 15–26/min	*p* = 0.638
	Temperature (On discharge; Recovered cases)	99.98 ± 1.664; 98–104°F	100.3 ± 1.746; 97.5–103.6°F	*p* = 0.252
Treatment outcomes among the study groups.	Recovered	*N* = 75 (74.25%)	*N* = 72 (69.23%)	
		Male - 55 (73.3%)	Male - 60 (83.3%)	
		Female - 20 (26.7%)	Female - 12(16.7%)	
	Dead	*N* = 26 (25.74%)	*N* = 32 (30.76%)	
		Male - 21 (%);	Male - 20 (62.5%)	
		Female - 5 (%)	Female - 12 (37.5%)	
	Time to Clinical Improvement	9.41 ± 5.38; 3–32 days	14.21 ± 5.694; 6–28 days	*p* = <0.0001****
	CT difference% (On admission and before discharge)	22.13 ± 9.662; 5–50%	11.74 ± 8.583; 0–35%	*p* = <0.0001****
	Time to symptomatic recovery	9.41 ± 5.38; 3–32 days	14.21 ± 5.694; 6–28 days	*p* = <0.0001****
	duration of ICU stay	7.68 ± 5.45; 1–27 days	10.59 ± 5.453; 2–42 days	*p* = 0.004**
	Total duration of Hospitalization	10.02 ± 6.277; 1–35 days	14.48 ± 8.882; 3–42 days	*p* = <0.0001****
	Duration of Hospitalization (Recovered patients)	11.09 ± 6.039; 3–35 days	16.31 ± 6.148; 7–30 days	*p* = <0.0001****
	Duration of ICU stay (Recovered patients)	7.947 ± 5.26; 1–26 days	10.72 ± 6.365; 2–26 days	*p* = 0.0045**
	Time to Clinical failure/death	6.88 ± 6.139; 1–27 days	10.38 ± 12.27; 3–42 days	*p* = 0.1986

Group A showed a better recovery rate of 74.25% and a lower death rate of 25.74% than that of group B, 69.23%, and 30.76%, respectively. The mean value of the time to clinical improvement (Group A 9.41; Group B 14.21 days), National Early Warning Score-2 on discharge (Group A 0.89; Group B 1.2), duration of ICU stay (Group A-7.68; Group B-10.58), and the total hospitalization duration (Group A 9.91; Group B 14.68) were less in the remdesivir–tocilizumab treatment group. Among the survivors of our study population, the mean duration of ICU (Group A 7.947; Group B 10.72) stay and the duration of hospitalization (Group A-11.09; Group B-16.31) were found lower in group A. A superior improvement of pulmonary pathology in the CT chest findings was observed (difference of involvement between the admission and discharge) among the survivors (Group A-22.13; Group B-11.74) who received remdesivir–tocilizumab as a treatment. All these differences were statistically significant in a *t*-test *p*=<0.05. Among the study groups, higher CT chest involvement (group A-47.33%; group-B 32.15%), P:F ratio (group A-90.48; group B-75.63), SOFA day-1 score (group-A 6.06; group-B 5.35), and low PaO_2_ (group A-46.09; group B-47.91) were observed during admission in group A, which differs significantly with those of the control group B, *p* = <0.05. –Table 1.

In all 60% of women in group A had a relatively shorter ICU stay (≤10 days) than that of the men. This was reversed in group B, where 66.25% of men recovered from the ICU within 10 days in comparison to 50% of women. In group A, both genders had almost similar duration of hospital stay, but men had a higher recovery period than women in group B, 4.8% (Stasi et al., 2020); patients in group B required >31 day of hospitalization. This was 0.09% (Velavan and Meyer, 2020) in group A. A large number of patients, 51.4%52), in Group A recovered within 10 days, compared to fewer, 19.2% (Roostaei Firozabad et al., 2021), in group B. In group A, 80% (Abbasajankea et al., 2021) of the females recovered within 10 days. This number was greater than that of the males, 64.45% (Annamaria et al., 2020). In group B, 33.3% (Roostaei Firozabad et al., 2021) of men achieved clinical improvement within 10 days, while no women recovered within this period. Among the survivors of group A, 41.5% (56.36% of men and 55% of thewomen) recovered within a period of 10 days. This was only 11.5% (20% of the men) in group B. Time to clinical failure/death was relatively fast in group B. –Table 2.

**TABLE 2:** Subgroup analysis according to the gender and the duration of COVID-19 illness.

Variables	Duration	Male		Female	
Group A, ICU stay	≤10 days	40 (52.6%)	*n* = 76	15 (60%)	*n* = 25
	11–20 days	33 (43.4%)		10 (40%)	
	21–30 days	3 (3.9%)		0	
	≥31 days	0		0	
Group B, ICU stay	≤10 days	53 (66.25%)	*n* = 80	12 (50%)	*n* = 24
	11–20 days	17 (21.25%)		8 (33.3%)	
	21–30 days	1 (1.25%)		0	
	≥31 days	1 (1.25%)		4 (16.6%)	
Group A (*n* = 101), Duration of total hospital stay	≤10 days	48 (63.15%)	*n* = 76	16 (64%)	*n* = 25
	11–20 days	20 (26.3%)		7 (28%)	
	21–30 days	7 (9.2%)		2 (8%)	
	≥31 days	1 (1.3%)		0	
Group B (*n* = 104), Duration of total hospital stay	≤10 days	29 (36.25%)	*n* = 80	8 (33.3%)	*n* = 24
	11–20 days	41 (51.25%)		8 (33.3%)	
	21–30 days	9 (11.25%)		4 (16.6%)	
	≥31 days	1 (1.25%)		4 (16.6%)	
Group A (*n* = 75); Time to Clinical Improvement.	≤10 days	36 (64.45%)	*n* = 55	16 (80%)	
	11–20 days	17 (30.9%)		03 (15%)	*n* = 20
	21–30 days	02 (3.6%)		01 (5%)	
Group B (*n* = 72); Time to Clinical Improvement	≤10 days	20 (33.3%)	*n* = 60	0	*n* = 12
	11–20 days	33 (55%)		8 (66.6%)	
	21–30 days	7 (11.6%)		4 (33.3%)	
Group A (*n* = 75), Recovered cases.Duration of ICU stay	≤10 days	31 (56.36%)	*n* = 55	11 (55%)	*n* = 20
	11–20 days	17 (30.9%)		7 (35%)	
	21–30 days	7 (12.7%)		2 (1%)	
	≥31 days	0		0	
Group B (*n* = 72), Recovered cases. Duration of ICU stay	≤10 days	12 (20%)	*n* = 60	0	*n* = 12
	11–20 days	40 (66.6%)		8 (66.6%)	
	21–30 days	8 (13.3%)		4 (33.3%)	
	≥31 days	0		0	
Group A (*n* = 26), Expired cases. Time to clinical failure/death	≤10 days	19 (90.4%)	*n* = 21	3 (60%)	*n* = 5
	11–20 days	1 (4.76%)		2 (40%)	
	21–30 days	1 (4.76%)		0	
Group B (*n* = 32), Expired cases. Time to clinical failure/death	≤10 days	19 (95%)	*n* = 20	9 (75%)	*n* = 12
	11–20 days	1 (5%)		3 (25%)	

### Survival Analysis Among the Treatment Groups

**FIGURE 2:**
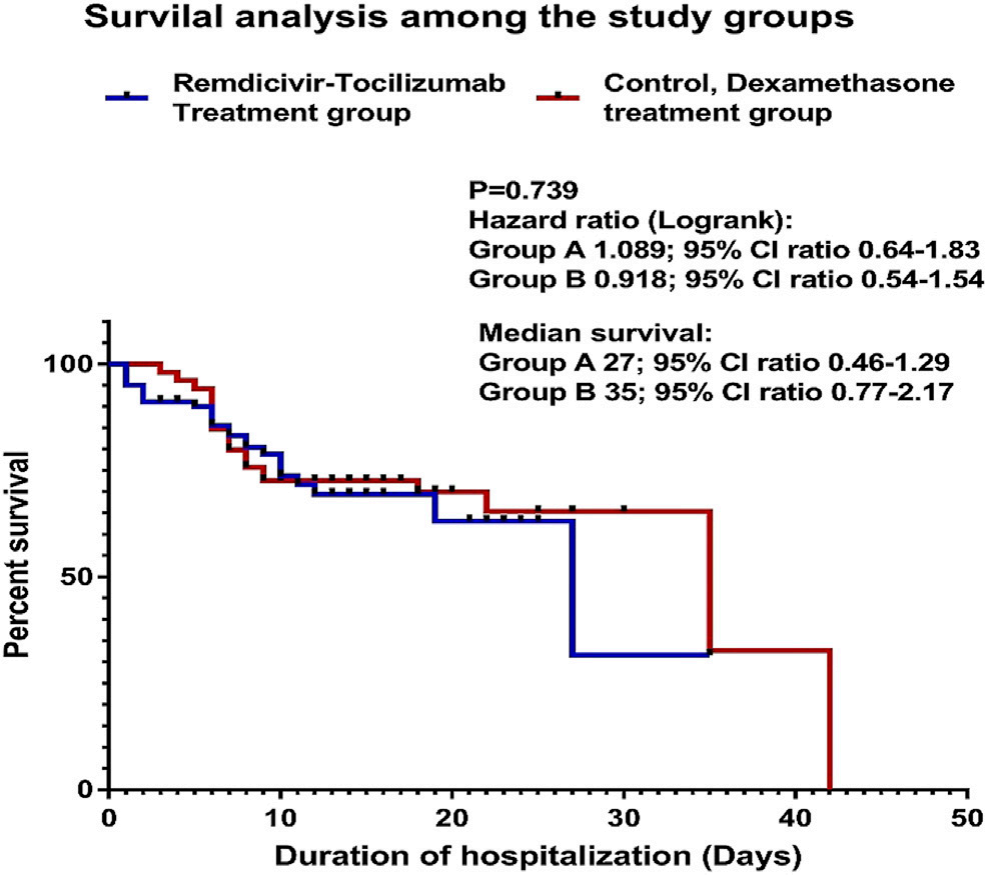
Kaplan–Meier survival analysis among the study groups.

The Kaplan–Meier survival analysis was performed. The difference between the two study groups was not significant, *p* = 0.739. hazard ratio (log-rank): group A (remdesivir–tocilizumab) 1.089; 95% CI ratio 0.64–1.83; group B (control/dexamethasone) 0.918; 95% CI ratio 0.54–1.54. median survival: Group A 27; 95% CI ratio 0.46–1.29; group B 35; 95% CI ratio 0.77–2.17 (Figure 2).

### Subgroup Analysis of the Total Duration of Hospitalization and ICU Stay Against the Age Group

Group A had a shorter duration of hospital stay and faster recovery. Of the total group A patients, 63.4% were discharged from the hospital within 10 days, and only 0.9% required >31 day; compared to 35.6 and 4.8% in group B. Regarding the ICU stay, Group B patients showed a faster recovery rate, 62.5%, within a period of 10 days than the 54.5% rate in group A. Though 8.6% of patients in group B required >31 days of ICU stay, no patients in group A required >31 days of ICU stay –Table 3.

**TABLE 3:** Subgroup analysis of the total duration of hospitalization and ICU stay against the age group.

Variables	Age group	Group A (Remdicivir-Tocilizumab)				Group B (Control; Dexamethasone)			
		1–10 days	11–20 days	21-30 days	>31 days	1–10 days	11–20 days	21–30 days	>31 days
Duration of Hospital Stay; Group A: *n* = 101; Group B, *n* = 104	11–20 years	4	1	0	0	0	0	0	0
	21–30 years	3	2	0	0	3	0	0	1
	31–40 years	6	2	0	0	8	8	0	0
	41–50 years	13	4	2	0	10	8	1	1
	51–60 years	16	4	5	0	4	8	4	0
	61–70 years	13	9	2	1	1	16	4	3
	71–80 years	7	4	0	0	11	5	4	0
	>81 years	2	1	0	0	0	4	0	0
Total		64 (63.4%)	27 (26.7%)	9 (8.9%)	1 (0.9%)	37 (35.6%)	49 (47.1%)	13 (12.5%)	5 (4.8%)
Duration of ICU Stay; Group A: *n* = 101; Group B, *n* = 104	11–20 years	3	2	0	0	0	0	0	0
	21–30 years	3	2	0	0	3	0	0	1
	31–40 years	5	3	0	0	12	4	0	0
	41–50 years	10	9	0	0	18	0	1	1
	51–60 years	13	10	2	0	12	0	4	0
	61–70 years	14	10	1	0	9	12	0	3
	71–80 years	6	5	0	0	11	5	4	0
	>81 years	1	2	0	0	0	4	0	0
Total		55 (54.5%)	43 (42.5%)	3 (2.9%)	0 (0%)	65 (62.5%)	25 (24%)	9 (8.6%)	5 (4.8%)

### The Subgroup Analysis of the Study Population Regarding Age Group

Full recovery was observed in the 11–20-years age group (5/5) in group A and the >81 years age group (4/4) in group B. The 21–30 years age group had a high mortality rate in both study groups, 60% in group A and 100% in group B. The 41–70 years age group had the highest number of hospital recoveries in group A, and the 31–70 years age group had the highest number of hospital recoveries in group B. Mortality was observed among the 51–60 years age group in group A and the 41–50 and 61–70 years age group in group B.–Table 4.

**TABLE 4:** Subgroup analysis of the study group patients according to the age.

Age group	Over all		Recovered patients		Dead cases	
	Group A	Group B	Group A	Group B	Group A	Group B
11–20 years	5 (5%)	0 (0%)	5 (6.6%)	0 (0%)	0 (0%)	0 (0%)
21–30 years	5 (5%)	4 (3.8%)	4 (5.3%)	0 (0%)	3 (11.5%)	4 (12.55)
31–40 years	8 (7.9%)	16 (15.38%)	5 (6.6%)	16 (22.2%)	3 (11.5%)	0 (0%)
41–50 years	19 (18.8%)	20 (19.23%)	16 (21.3%)	8 (11.1%)	6 (23%)	12 (37.5%)
51–60 years	25 (24.8%)	16 (15.38%)	19 (25.3%)	16 (22.2)	8 (30.7%)	4 (12.5%
61–70 years	25 (24.8%)	24 (23.0%)	17 (22.6%)	20 (27.7%)	4 (15.4%)	12 (37.5%)
71–80 years	11 (10.9%)	20 (19.23%)	7 (9.3%)	8 (11.1%)	1 (3.8%)	0 (0%)
>81 years	3 (3.0%)	4 (3.8%)	2 (2.6%)	4 (5.5%)	1 (3.8%)	0 (0%)
Total	101 (100%)	104 (100%)	75 (100%)	72 (100%)	26 (100%)	32 (100%)

### Subgroup Analysis of the Recovered and Death Cases Against Duration and Age

The early and the late age group of 11–40 years and 71->81 years, respectively, had a 100% hospital recovery within a 20 days period in group A. The same was observed among the 21–50 years and >81 years age groups in group B. Hospital recovery time was prolonged to >21 days among the 41–70 years age group in group A and 51–80 years age group in group B. Up to 80% hospital recovery was observed among all the age groups of group A within 10 days. On the other hand, most of the patients in group B were discharged from the hospital within 11–20 days. Most of the patients in all ages of group A recovered from the ICU within 10 days. On the contrary, the highest recoveries were seen within 11–20 days in group B. Group B had higher mortality (87.5%) than group A (61.5%) within 10 days –Table 5.

**TABLE 5:** Analysis of the recovered and expired COVID-19 cases against recovery duration /death and age.

Variables	Age group	Group A (Remdicivir-Tocilizumab)					Group B (Control; Dexamethasone)				
		1–10 days	11–20 days	21–30 days	>31 days	Total	1–10 days	11–20 days	21–30 days	>31 days	Total
Duration of Hospital Stay (Recovered cases); Group A, *n* = 75; Group B, *n* = 72	11–20 years	4	1	0	0	5	0	0	0	0	0
	21–30 years	2	2	0	0	4	0	0	0	0	0
	31–40 years	3	2	0	0	5	8	8	0	0	16
	41–50 years	10	4	2	0	16	0	8	0	0	8
	51–60 years	11	4	4	0	19	4	8	4	0	16
	61–70 years	7	7	2	1	17	0	16	4	0	20
	71–80 years	4	3	0	0	7	0	4	4	0	8
	>81 years	1	1	0	0	2	0	4	0	0	4
	Total	42 (56%)	24 (32%)	8 (10.6%)	1 (1.3%)	75 (100%)	12 (16.7%)	48 (66.7%)	12 (16.7%)	0 (0%)	72 (100%)
Duration of ICU Stay (Recovered cases); Group A, *n* = 75; Group B, *n* = 72	11–20 years	4	1	0	0	5	0	0	0	0	0
	21–30 years	3	1	0	0	4	0	0	0	0	0
	31–40 years	5	0	0	0	5	12	4	0	0	16
	41–50 years	12	4	0	0	16	8	0	0	0	8
	51–60 years	14	4	1	0	19	12	0	4	0	16
	61–70 years	11	5	1	0	17	8	12	0	0	20
	71–80 years	6	1	0	0	7	0	4	4	0	8
	>81 years	2	0	0	0	2	0	4	0	0	4
	Total	57 (76%)	16 (21.3%)	2 (2.6%)	0 (0%)	75 (100%)	40 (55.5%)	24 (33.3%)	8 (11.1%)	0 (%)	72 (100%)
Duration of Hospital/ICU Stay (Death cases); Group A, *n* = 26; Group B *n* = 32	11–20 years	0	0	0	0	0	0	0	0	0	0
	21–30 years	1	0	0	0	1	4	0	0	0	4
	31–40 years	2	1	0	0	3	0	0	0	0	0
	41–50 years	1	2	0	0	3	10	0	0	2	12
	51–60 years	4	1	1	0	6	4	0	0	0	4
	61–70 years	6	2	0	0	8	10	1	1	0	12
	71–80 years	1	3	0	0	4	0	0	0	0	0
	>81 years	1	0	0	0	1	0	0	0	0	0
	Total	16 (61.5%)	9 (34.6%)	1 (3.8%)	0 (0%)	26 (100%)	28 (87.5%)	1 (3.1%)	1 (3.1%)	2 (6.3%)	32 (100%)

### Distribution and Subgroup Analysis of Comorbidity Among the Study Groups

It was found that 58.7%61) patients in group A and 44.2%44) patients in group B had comorbid conditions. They were hypertension (HTN), diabetes mellitus (DM), ischemic heart disease (IHD), chronic obstructive pulmonary airway disease (COPD), rheumatoid arthritis (RA), benign prostatic hyperplasia (BPH), osteoarthritis, hypothyroid, ischemic stroke, heart failure, chronic kidney disease, bronchial asthma, inflammatory bowel disease (IBD), irritable bowel syndrome (IBS), hepatitis B, migraine, and early carcinoma. Nearly similar numbers of comorbidities were noted in both study groups; 15.8% (Abbasajankea et al., 2021) and 21.1% (Cao et al., 2020) had HTN in groups A and B, respectively. A better percentage of recovery was achieved by the group A patients who had comorbidities with HTN than that of group B. Similarly, the percentage of death was high among those in group B who had HTN or DM and other comorbidities than in group A. In addition, in group A, diabetic patients with comorbidities experienced better recovery. –Table 6.

**TABLE 6:** Distribution and analysis of comorbidity among the study group patients.

Comorbidity	Group A. Male 46 (75.4%); Female 15 (24.59%)			Group B. Male 33 (71.7%); Female 13(28.3%)		
	Total 61 (58.7%)	Recovered 21 (34.42%)	Death Cases 15 (24.59%)	Total 46 (44.2%)	Recovered 24 (52.1%)	Death Cases 10 (21.7%)
HTN	16 (15.8%)	11 (68.75%)	5 (31.2%)	22 (21.1%)	14 (%)	8 (%)
IHD	8 (7.9%)	3 (37.5%)	5 (62.5%)	3 (2.8%)	3 (100%)	0
Diabetes Mellitus	15 (24.5%)	7 (46.7%)	8 (53.3%)	13 (12.5%)	6 (46.2%)	7 (53.8%)
COPD	5 (4.8%)	2 (40%)	3 (60%)	4 (3.9%)	4 (100%)	0
BPH	9 (8.9%)	5 (55.6%)	4 (44.4%)	3 (2.8%)	1 (33.3%)	2 (66.7%)
Rheumatoid Arthritis	4 (3.8%)	4 (100%)	0	2 (1.9%)	2 (100%)	0
Osteoarthritis	5 (4.8%)	5 100(%)	0	1 (0.9%)	1 (100%)	0
Hypothyroid	3 (2.9%)	3 (100%)	0	1 (0.9%)	1 (100%)	0
Ischemic stroke	2 (1.9%)	2 (%)	0	1 (0.9%)	1 (7.1%)	0
Heart failure	2 (1.9%)	1 (50%)	1 (50%)	2 (1.9%)	0	2 (100%)
Chronic Kidney Disease	1 (1%)	0	1 (100%)	1 (0.9%)	1 (100%)	0
Bronchial Asthma	1 (1%)	1 (100%)	0	2 (1.9%)	0	2 (100%)
IBD	1 (1%)	0	1 (100%)	0 (0 %)	0	0
IBS	0	0	0	1 (0.9%)	1 (100%)	0
Hepatitis B	1 (1%)	1 (100%)	0	1 (0.9%)	1 (100%)	0
Migraine	1 (1%)	1 (100%)	0	3 (2.8%)	1 (33.3%)	2 (66.7%)
Carcinoma (Early)	1 (1%)	1 (100%)	0	0	0	0

## Discussion

In this research, a multicenter study of 205 selected patients after exclusion was carried out in the COVID-19 special care and dependency unit of multiple health centers/hospitals in Bangladesh. Combination therapy of remdesivir with tocilizumab was carried out in the intervention group and was compared with that in the control group which was treated with dexamethasone. To evaluate equal treatment outcomes among the study groups, COVID-19 cases in ventilator support and with severe comorbidities were excluded. The post-intervention outcome was measured as described in the methodology. Kaplan-Meier survival analysis was performed, and no significant advantage of the remdesivir–tocilizumab treatment was observed against dexamethasone therapy, *p* = 0.739. However, the remdesivir–tocilizumab intervention group had a relatively lower mortality rate of 25.74% than that of the control group, which was 30.76%, in our study. There was significant reduction in the time to clinical improvement, NEWS-2 score on discharge, CT improvement (%), and duration of ICU and hospital stays in the remdesivir–tocilizumab group, *p*=<0.05 (Table 1). This reduction was equally observed among the patients with comorbidities. Patients with multiple comorbidities in Group A also had better prognosis and low mortality than those in the control group (Table 6). Therefore, the remdesivir–tocilizumab combination was found to have a favorable treatment outcome compared to dexamethasone therapy in case of severe COVID-19 concerning the mortality rate, time to clinical improvement, duration of ICU stay, and the total duration of hospitalization.

Currently, many trials are ongoing for the management of COVID-19, and several trials have been completed recently. (WHO Solidarity Trial Consortium PHPRea, 2021; Tabarsi et al., 2021; Abbasajankea et al., 2021; Les bujandai lajbfea et al., 2021; Dabbousesmeagea et al., 2021; Reis et al., 2021; Roostaei Firozabad et al., 2021; Granholm et al., 2021) Among these clinical trials, several discussed the repurposing of the approved drugs for the management of COVID-19. Possible therapeutic agents such as different antiviral drugs, existing drugs that have an antiviral effect, corticosteroids, and other possible agents are being investigated. A randomized controlled trial on the lopinavir/ritonavir therapy against the standard care in China has revealed no benefit against the standard management. (Cao et al., 2020) Corticosteroids, for example, dexamethasone and methylprednisolone have been suggested and used with patients of severe illness to prevent the overactive immune system and cytotoxic storm. Though, the improvement of COVID-19 patients mostly depends on disease severity, the age, existing comorbidity, and the physical status of the affected patient (Liu and Liu, 2020b), several studies have shown that remdesivir and tocilizumab clinically benefit patients with moderate-to-severe COVID-19, but none of these studies examined a combination of the two therapies (Wangzddgea et al., 2020; Singh et al., 2020; Cao et al., 2020; Greinonsdea et al., 2020; Xuhmltea et al., 2020; Stonefmsbnea et al., 2020; Lan et al., 2020). Thus, it was necessary to investigate these two drugs in combination for the treatment of severe COVID-19. As an antiviral agent, remdesivir was found to be beneficial for the management of COVID-19 (Wangzddgea et al., 2020; Singh et al., 2020). Similarly, the role of tocilizumab was also found preferable in multiple published studies (Xuhmltea et al., 2020; Stonefmsbnea et al., 2020; Lan et al., 2020). We also found comparable outcomes in our current study. Therefore, the remdesivir–tocilizumab combination might be a preferable choice of treatment for the management of severe COVID-19. Further study with a larger sample size is necessary to solidify the outcome of this combination.

Our study has limitations: the small sample size; exclusion of COVID-19 cases that required ventilator support, and COVID-19 patients with severe comorbidities are a concern. In addition, analysis depending on the severity of COVID-19–ARDS was not performed. But to the best of our effort, we selected severe COVID-19–ARDS cases devoid of serious or uncontrolled comorbidity to ensure proper comparison and outcome among the study groups without influence.

## Conclusion

According to this study, the remdesivir–tocilizumab combination had preferable outcomes compared to those of the dexamethasone therapy for the treatment of severe COVID-19 concerning the lower mortality rate, better clinical and pulmonary improvement, reduced duration of the ICU stay, and decreased hospitalization period, although the survival benefit of this combination therapy was not significant against dexamethasone alone.

## Data Availability

The raw data supporting the conclusions of this article will be made available by the authors, without undue reservation.
