# Supplementary material for: Efficacy and Outcome of Remdesivir and Tocilizumab Combination Against Dexamethasone for the Treatment of Severe COVID-19: A Randomized Controlled Trial
Source: Front Pharmacol. 2022 Apr 5;13:690726. doi: 10.3389/fphar.2022.690726 (PMC9017992; doi:10.3389/fphar.2022.690726)
Supplement: Supplementary file 1 [file DataSheet1.pdf]

Table 1: Patient demographics, characteristics, and treatment outcomes among the study groups. Data presented as mean ± SD.

	Variables	Group A	Group B (Control)	T-test (95% CI)
Patient demographics and characteristics of study group patients during hospitalization.	Gender	n =101; Male 76 (73.1%); Female- 25 (24%)	n = 104; Male – 80 (76.93%); Female- 24(23.07%)	
	Age (In years) 56.64±15.05; 18-85years	56.24±15; 18-85years	57.04±15.15; 23-83years	P=0.259
	Body weight (Kg)	65.4± 9.0(51-94)	66.5± 7.7(54-91)	P=0.61
	BMI	23.6± 5.6 (19.8-36.4)	22.9± 5.8 (21.4-35.7)	P=0.58
	Comorbidity	61(58.7%)	46(44.2%)	
	NEWS-2 Score (On admission)	8.267±1.918; 5-12	8.788±2.037; 5-13	p=0.060
	SOFA (Sequential organ failure assessment) day-1 score	6.06±1.67; 3-11	5.35±1.58; 3-11	p=0.021**
	Oxygen saturation (%)	85.77±8.971; 34-99%	85.23±6.961; 73-98%	p=0.0243*
	PaO2 (mm of Hg)	46.09±9.8; 23-83	47.91±6.8; 38-91	p=0.005**
	P:F ratio (On admission)	90.48±39.6; 23-199	75.63±41.79; 38-220	p=0.0093**
	Oxygen requirement	19.44±16.56; 4-60 L/min	18.38±10.51; 2-40 L/min	p=0.643
	CT chest %	47.33±19.4; 15-95%	32.15±17.51; 0-65%	p=<0.0001****
	Respiratory rate	29.07±10; 12-55/min	30.37±6.865; 18-45/min	p=0.299
	Temperature	100.7±1.865; 98-104^{0}F	101.4±1.453; 98-104^{0}F	p=0.398
	Serum Creatinine	1.14±0.8; 0.8-3	0.98±0.7;0.6-3	p=0.118
	Serum Bilirubin	1.62±1.07; 0.4-5.3	1.52±0.9; 0.4-5	p=0.19
	Platelet count (10^3 /ml)	207.5±92.8; 75-400	224.4±91.2; 87-400	p=0.19
	MAP(Mean arterial pressure in mm of Hg)	86.16±13.55; 54-120	86.26±12.19; 54-116	p=0.95
	GCS Score	12.19±1.79; 7-15	12.67±1.93; 7-15	p=0.064
Characteristics of study group patients during discharge.	NEWS-2 Score (On discharge; Recovered cases)	0.89±0.84; 0-2	1.22±0.87; 0-2	p=0.0221*
	Oxygen saturation (On discharge; Recovered cases)	95.91±2.07; 90-100 %	96.35±1.558; 93-99%	p=0.080

(Recovered cases)	Oxygen requirement (On discharge; Recovered cases)	1.36±2.288; 0-10 L/min	1.569±2.42; 0-12 L/min	p=0.279
	CT chest % (On discharge; Recovered cases)	17.67±8.193; 0-35%	15.35±10.07; 0-38%	p=0.127
	Respiratory rate (On discharge; recovered cases)	19.4±4.74; 12-34/min	19.11±2.17; 15-26/min	p=0.638
	Temperature (On discharge; Recovered cases)	99.98±1.664; 98-104^{0}F	100.3±1.746; 97.5-103.6^{0}F	p=0.252
Treatment outcomes among the study groups.	Recovered	N= 75 (74.25%)	N= 72 (69.23%)	
		Male 55(73.3%);	Male -60 (83.3%)	
		Female 20(26.7%)	Female-12(16.7%)	
	Dead	N= 26 (25.74%)	N= 32 (30.76%)	
		Male - 21(%);	Male – 20 (62.5%)	
		Female-5 (%)	Female- 12(37.5%)	
	Time to Clinical Improvement	9.41±5.38; 3-32 days	14.21±5.694; 6-28 days	p=<0.0001****
	CT difference% (On admission and before discharge)	22.13±9.662; 5-50%	11.74±8.583; 0-35%	p=<0.0001****
	Time to symptomatic recovery	9.41±5.38; 3-32 days	14.21±5.694; 6-28 days	p=<0.0001****
	duration of ICU stay	7.68±5.45; 1-27 days	10.59± 5.453; 2-42 days	p=0.004**
	Total duration of Hospitalization	10.02±6.277; 1-35 days	14.48±8.882; 3-42 days	p=<0.0001****
	Duration of Hospitalization (Recovered patients)	11.09±6.039; 3-35days	16.31±6.148; 7-30days	p=<0.0001****
	Duration of ICU stay (Recovered patients)	7.947±5.26; 1-26days	10.72±6.365; 2-26days	p=0.0045**
	Time to Clinical failure/death	6.88±6.139; 1-27 Days	10.38±12.27; 3-42 Days	p=0.1986

Table 2: Subgroup analysis according to the gender and the duration of COVID-19 illness.

Variables	Duration	Male		Female	
Group A, ICU stay	≤10days	40(52.6%)		15(60%)	
	11-20days	33(43.4%)	n=76	10(40%)	n=25
	21-30days	3(3.9%)		0	
	≥31days	0		0	
Group B, ICU stay	≤10days	53(66.25%)		12(50%)	
	11-20days	17(21.25%)	n=80	8(33.3%)	n=24
	21-30days	1(1.25%)		0	
	≥31days	1(1.25%)		4(16.6%)	
Group A (n=101), Duration of total hospital stay	≤10days	48(63.15%)		16(64%)	
	11-20days	20(26.3%)	n=76	7(28%)	n=25
	21-30days	7(9.2%)		2(8%)	
	≥31days	1(1.3%)		0	
Group B (n=104,)Duration of total hospital stay	≤10days	29(36.25%)		8(33.3%)	
	11-20days	41(51.25%)	n=80	8(33.3%)	n=24
	21-30days	9(11.25%)		4(16.6%)	
	≥31days	1(1.25%)		4(16.6%)	
Group A (n=75); Time to Clinical Improvement.	≤10days	36(64.45%)		16(80%)	
	11-20days	17(30.9%)	n=55	03(15%)	n=20
	21-30days	02(3.6%)		01(5%)	
Group B (n=72); Time to Clinical Improvement	≤10days	20(33.3%)		0	
	11-20days	33(55%)	n=60	8(66.6%)	n=12
	21-30days	7(11.6%)		4(33.3%)	
Group A (n=75), Recovered cases. Duration of ICU stay	≤10days	31(56.36%)		11(55%)	
	11-20days	17(30.9%)	n=55	7(35%)	n=20
	21-30days	7(12.7%)		2(1%)	
	≥31days	0		0	
Group B (n=72), Recovered cases. Duration of ICU stay	≤10days	12(20%)		0	
	11-20days	40(66.6%)	n=60	8(66.6%)	n=12
	21-30days	8(13.3%)		4(33.3%)	
	≥31days	0		0	
Group A (n=26), Expired cases. Time to clinical failure/death	≤10days	19(90.4%)		3(60%)	
	11-20days	1(4.76%)	n=21	2(40%)	n=5
	21-30days	1(4.76%)		0	
Group B (n=32), Expired cases. Time to clinical failure/death	≤10days	19(95%)		9(75%)	
	11-20days	1(5%)	n=20	3(25%)	n=12

Table: 3 Subgroup analysis of the total duration of hospitalization and ICU stay against the age group.

Variables	Age group	Group A (Remdicivir-Tocilizumab)				Group B (Control; Dexamethasone)			
		1-10days	11-20days	21-30 days	>31 days	1-10 days	11-20 days	21-30 days	>31 days
Duration	11-20 yrs	4	1	0	0	0	0	0	0
of	21-30 yrs	3	2	0	0	3	0	0	1
Hospital	31-40 yrs	6	2	0	0	8	8	0	0
Stay	41-50 yrs	13	4	2	0	10	8	1	1
Group A	51-60 yrs	16	4	5	0	4	8	4	0
n=101,	61-70 yrs	13	9	2	1	1	16	4	3
Group B	71-80 yrs	7	4	0	0	11	5	4	0
n=104	>81 yrs	2	1	0	0	0	4	0	0
Total		64(63.4%)	27(26.7%)	9(8.9%)	1(0.9%)	37(35.6%)	49(47.1%)	13(12.5%)	5(4.8%)
	11-20 yrs	3	2	0	0	0	0	0	0
Duration of	21-30 yrs	3	2	0	0	3	0	0	1
ICU Stay	31-40 yrs	5	3	0	0	12	4	0	0
Group A	41-50 yrs	10	9	0	0	18	0	1	1
n=101,	51-60 yrs	13	10	2	0	12	0	4	0
Group B	61-70 yrs	14	10	1	0	9	12	0	3
n=104	71-80 yrs	6	5	0	0	11	5	4	0
	>81 yrs	1	2	0	0	0	4	0	0
Total		55(54.5%)	43(42.5%)	3(2.9%)	0(0%)	65(62.5%)	25(24%)	9(8.6%)	5(4.8%)

Table 4: Subgroup analysis of the study group patients according to the age.

Age group	Over all		Recovered patients		Dead cases	
	Group A	Group B	Group A	Group B	Group A	Group B
11-20 yrs	5(5%)	0(0%)	5(6.6%)	0(0%)	0(0%)	0(0%)
21-30 yrs	5(5%)	4(3.8%)	4(5.3%)	0(0%)	3(11.5%)	4(12.55)
31-40 yrs	8(7.9%)	16(15.38%)	5(6.6%)	16(22.2%)	3(11.5%)	0(0%)
41-50 yrs	19(18.8%)	20(19.23%)	16(21.3%)	8(11.1%)	6(23%)	12(37.5%)
51-60 yrs	25(24.8%)	16(15.38%)	19(25.3%)	16(22.2)	8(30.7%)	4(12.5%
61-70 yrs	25(24.8%)	24(23.0%)	17(22.6%)	20(27.7%)	4(15.4%)	12(37.5%)
71-80 yrs	11(10.9%)	20(19.23%)	7(9.3%)	8(11.1%)	1(3.8%)	0(0%)
>81 yrs	3(3.0%)	4(3.8%)	2(2.6%)	4(5.5%)	1(3.8%)	0(0%)
Total	101(100%)	104(100%)	75(100%)	72(100%)	26(100%)	32(100%)

Table 5: Analysis of the recovered and expired COVID-19 cases against recovery duration /death and age.

Variables	Age group	Group A (Remdicivir-Tocilizumab)					Group B (Control; Dexamethasone)				
		1-10days	11-20days	21-30 days	>31 days	Total	1-10 days	11-20 days	21-30 days	>31 days	Total
Duration of	11-20 yrs	4	1	0	0	5	0	0	0	0	0
Hospital Stay	21-30 yrs	2	2	0	0	4	0	0	0	0	0
(Recovered	31-40 yrs	3	2	0	0	5	8	8	0	0	16
cases)	41-50 yrs	10	4	2	0	16	0	8	0	0	8
Group A n=75	51-60 yrs	11	4	4	0	19	4	8	4	0	16
Group B n=72	61-70 yrs	7	7	2	1	17	0	16	4	0	20
	71-80 yrs	4	3	0	0	7	0	4	4	0	8
	>81 yrs	1	1	0	0	2	0	4	0	0	4
	Total	42(56%)	24(32%)	8(10.6%)	1(1.3%)	75(100%)	12(16.7%)	48 (66.7%)	12(16.7%)	0(0%)	72(100%)
Duration of	11-20 yrs	4	1	0	0	5	0	0	0	0	0
ICU Stay	21-30 yrs	3	1	0	0	4	0	0	0	0	0
(Recovered cases)	31-40 yrs	5	0	0	0	5	12	4	0	0	16
Group A n=75	41-50 yrs	12	4	0	0	16	8	0	0	0	8
	51-60 yrs	14	4	1	0	19	12	0	4	0	16
Group B n=72	61-70 yrs	11	5	1	0	17	8	12	0	0	20
	71-80 yrs	6	1	0	0	7	0	4	4	0	8
	>81 yrs	2	0	0	0	2	0	4	0	0	4
	Total	57(76%)	16(21.3%)	2(2.6%)	0(0%)	75(100%)	40(55.5%)	24(33.3%)	8(11.1%)	0(%)	72(100%)
Duration of	11-20 yrs	0	0	0	0	0	0	0	0	0	0

Hospital/ICU Stay (Death cases) Group A n=26 Group B n=32	21-30 yrs	1	0	0	0	1	4	0	0	0	4
	31-40 yrs	2	1	0	0	3	0	0	0	0	0
	41-50 yrs	1	2	0	0	3	10	0	0	2	12
	51-60 yrs	4	1	1	0	6	4	0	0	0	4
	61-70 yrs	6	2	0	0	8	10	1	1	0	12
	71-80 yrs	1	3	0	0	4	0	0	0	0	0
	>81 yrs	1	0	0	0	1	0	0	0	0	0
	Total	16(61.5%)	9(34.6%)	1(3.8%)	0(0%)	26(100%)	28(87.5%)	1(3.1%)	1(3.1%)	2(6.3%)	32(100%)

Table 6: Distribution and analysis of comorbidity among the study group patients.

Comorbidity	Group A. Male 46(75.4%) Female 15 (24.59%)			Group B. Male 33(71.7%), Female 13(28.3%)		
	Total 61(58.7%)	Recovered 21(34.42%)	Death Cases 15(24.59%)	Total 46(44.2%)	Recovered 24(52.1%)	Death Cases 10(21.7%)
HTN	16 (15.8%)	11(68.75%)	5(31.2%)	22(21.1%)	14(%)	8(%)
IHD	8(7.9%)	3(37.5%)	5(62.5%)	3(2.8%)	3(100%)	0
Diabetes Mellitus	15(24.5%)	7(46.7%)	8(53.3%)	13(12.5%)	6(46.2%)	7(53.8%)
COPD	5(4.8%)	2(40%)	3(60%)	4(3.9%)	4(100%)	0
BPH	9(8.9%)	5(55.6%)	4(44.4%)	3(2.8%)	1(33.3%)	2(66.7%)
Rheumatoid Arthritis	4(3.8%)	4(100%)	0	2(1.9%)	2(100%)	0
Osteoarthritis	5(4.8%)	5100(%)	0	1(0.9%)	1(100%)	0
Hypothyroid	3(2.9%)	3(100%)	0	1(0.9%)	1(100%)	0
Ischemic stroke	2(1.9%)	2(%)	0	1(0.9%)	1(7.1%)	0
Heart failure	2 (1.9%)	1(50%)	1(50%)	2(1.9%)	0	2(100%)
Chronic Kidney Disease	1(1%)	0	1(100%)	1(0.9%)	1(100%)	0
Bronchial Asthma	1(1%)	1(100%)	0	2(1.9%)	0	2(100%)
IBD	1(1%)	0	1(100%)	0(0 %)	0	0
IBS	0	0	0	1(0.9%)	1(100%)	0
Hepatitis B	1(1%)	1(100%)	0	1(0.9%)	1(100%)	0
Migraine	1(1%)	1(100%)	0	3(2.8%)	1(33.3%)	2(66.7%)
Carcinoma (Early)	1(1%)	1100%)	0	0	0	0
